# Visual analogue scale for sino-nasal symptoms severity correlates with sino-nasal outcome test 22: paving the way for a simple outcome tool of CRS burden

**DOI:** 10.1186/s13601-018-0219-6

**Published:** 2018-09-03

**Authors:** Maria Doulaptsi, Emmanuel Prokopakis, Sven Seys, Benoit Pugin, Brecht Steelant, Peter Hellings

**Affiliations:** 1 Laboratory of Clinical Immunology, Department of Microbiology and Immunology, KU Leuven, Kapucijnenvoer 33, 3000 Louvain, Belgium; 2 Department of Otorhinolaryngology, Head and Neck Surgery, University of Crete School of Medicine, Heraklion, Crete, Greece; 3 Clinical Division of Otorhinolaryngology, Head and Neck Surgery, University Hospitals Leuven, Louvain, Belgium

**Keywords:** Chronic rhinosinusitis, Patient reported outcome measures, Quality of life, SNOT-22, VAS

## Abstract

**Background:**

A visual analogue scale (VAS) is a psychometric instrument widely used in the Rhinology field to subjectively quantify patient’s symptoms severity. In allergic rhinitis, VAS has been found to correlate well with the allergic rhinitis and its impact on asthma severity classification, as well as with rhinoconjunctivitis quality of life questionnaire. In chronic rhinosinusitis (CRS), total VAS score are often used to classify disease burden into mild, moderate, and severe, with few studies correlating VAS scores with more complex and validated instruments assessing disease-specific burden like Sino-Nasal Outcome Test (SNOT)-22.

**Methods:**

We correlated VAS scores for total and individual sino-nasal symptom with SNOT-22 scores in a randomly selected group of 180 CRS patients. Pearson’s rho was selected as a correlation coefficient for analysis.

**Results:**

VAS scores for total nasal symptom score and individual symptoms correlated significantly with SNOT-22, irrespective of VAS based subclasses for sino-nasal, ocular, and bronchial symptoms.

**Conclusions:**

VAS for total sino-nasal symptom severity might be used for assessing disease severity, monitoring the course of the disease, and can be used for treatment decisions and disease burden.

**Electronic supplementary material:**

The online version of this article (10.1186/s13601-018-0219-6) contains supplementary material, which is available to authorized users.

## Background

Chronic rhinosinusitis (CRS), with or without nasal polyps, is defined as an inflammation of nose and paranasal sinuses lasting for at least 12 weeks [1]. It is characterized by two or more symptoms, one of which should be either nasal blockage/obstruction or nasal secretions (anterior/posterior nasal drip). Other symptoms might be facial pain/pressure and hyposmia/anosmia. The prevalence of CRS in the European adult population is estimated by GA(2)LEN study to be around 11.9%, while in the USA it is considered even higher [2, 3]. The burden of disease and the impact on patients’ every day activity, work productivity, and overall Quality of Life (QoL) cannot be underestimated, especially in difficult-to-treat cases [4]. The direct cost of CRS in the United States is estimated at $8.6 billion/year, while societal indirect costs from productivity loss are approximately $10,077 per patient each year [5]. Interestingly, general health of CRS patients was worse compared to patients with congestive heart failure, Chronic Obstructive Pulmonary Disease (COPD), and Parkinson disease, using generic health-state utility scores [6].

To accurately assess the burden of disease in CRS patients, multiple disease-specific QoL questionnaires were designed and validated over the past years [7–10]. These questionnaires focus on symptoms and how they affect patients’ daily life, emotional condition, and overall QoL. These instruments are designed to have a strong association with principal disease characteristics and the ability to reflect response to treatment. Among different disease-specific outcome measurements in CRS, the Sino-Nasal Outcome Test (SNOT)-22 is widely accepted and has been used in several studies even before its validation by Hopkins et al., in 2009. SNOT-22 is a reliable questionnaire, can be used to facilitate clinical practice, and validated in multiple languages [8].

Visual analogue scale (VAS) is a psychometric measurement instrument widely used in the Rhinology field and beyond to subjectively quantify patients’ symptoms severity. Originally designed to evaluate workers productivity by senior personnel, VAS gained more attention in the sixties in medicine, social science, and market research [11]. It represents a horizontal line of 10 cm with word anchors at each end representing the extreme feelings. Patients are instructed to indicate the point on the line that best corresponds to their status for the particular characteristic being evaluated. In addition to its high sensitivity, reliability and reproducibility, VAS is easy and simple to use by patients and health care providers [11]. It also does not require training, making VAS a highly valuable tool not only for everyday clinical practice, but also for real-life studies [11].

In allergic disease, VAS was found to correlate well with the Allergic Rhinitis and its Impact on Asthma (ARIA) severity classification system and QoL measurement instruments such as Rhinoconjunctivitis Quality of Life Questionnaire (RQLQ) [12]. Additionally, VAS is utilized to monitor the course of the disease, to assess treatment outcomes, to obtain self-assessments, and to define the level of control in allergic patients by MACVIA-ARIA project [13]. Lately, VAS has been incorporated into the MASK Allergy Diary mobile app, a clinical decision support system assessing allergic rhinitis (AR) severity for feedback to the patient and the doctor on level of disease control [14].

In CRS, VAS for total nasal symptom score (TNSS) is part of routine clinical practice to classify disease as mild, moderate, and severe. In research, VAS for TNSS and individual symptoms are frequently incorporated into studies as an instrument for estimating symptoms severity and burden of disease [15]. In contrast to allergy, correlations between VAS scores with more complex instruments assessing disease-specific burden like SNOT-22 in CRS are scarce [16, 17]. Toma and Hopkins, demonstrated a strong correlation between VAS and SNOT-22 in 65 CRS patients and they further attempted to stratify SNOT-22 score based on disease severity [17]. Here, we aim to study VAS scores for TNSS and individual sino-nasal symptoms in relation to SNOT-22 scores in a larger randomly selected group of CRS patients. In addition, correlation between VAS and SNOT-22 scores is explored in different CRS phenotypes (with/without nasal polyps, controlled/partly controlled/uncontrolled disease).

## Methods

### Study population

A postal questionnaire survey was conducted at the Department of Otorhinolaryngology, Head and Neck Surgery of the University Hospitals of Leuven in Belgium. Subjects who visited the outpatient clinic and coded as CRS between January and May of 2016 were isolated from the clinical workstation. Evaluation of full medical records was performed by an ENT specialist to confirm the coded diagnosis based on EPOS defining criteria for CRS (symptoms, compatible endoscopic findings and/or computed tomography abnormalities when imaging was available). Patients younger than 16 years, those with primary immunodeficiency, ciliary dyskinesia, cystic fibrosis, malignant tumors, and/or surgery for CRS within 6 months were excluded from the study. Patients with a diagnosis of psychiatric disorder were also excluded from the study as their ability to give reliable information regarding their nasal disease could be disputed. Concomitant AR or bronchial asthma was not considered to be a limitation for participation in the study.

After evaluation, a questionnaire was sent to 325 consecutive subjects who met the above criteria. Patients received a postal questionnaire together with the study rationale, and an informed consent. Patients were asked to fill out the questionnaire after signing the informed consent and return all forms by post. In order to reach a response rate of 60%, some of the non-responders to our questionnaire were contacted by phone to communicate the importance of the study and encourage them to participate. This study has been approved by the institutional Medical Ethics Committee.

### Questionnaires

Subjects were asked to give information about their nasal disease, current and past medication for CRS, medical and surgical history, and to provide answers in general items such as profession and smoking habits. In another part of the questionnaire, patients had to give information regarding their allergic status, and their current medication for this condition. The same was asked in case of asthma comorbidity.

A special part of the questionnaire was dedicated to assessing patients’ QoL and severity of symptoms by using the SNOT-22 questionnaire and VAS scores. Patients had to draw a vertical line on a 10 cm scale from 0 to 10, according to “how bothersome your total nasal-sinus symptoms were within the last month” (see Additional file 1). Zero represented “not at all” and 10 represented “more than I can imagine” for TNSS. The same was asked for individual symptoms such as nasal blockage, headache/pain on the face, loss of smell, postnasal drip, runny nose, itchy nose, sneezing, itchy eyes, tearing, cough, tightness on the chest, shortness of breath and wheezing.

Level of control was assessed by using the following cut-off points for TNSS: well controlled (VAS ≤ 2), partly controlled (VAS > 2 and ≤ 5), uncontrolled (VAS > 5). In order to exclude a possible responder bias from the written questionnaires, 20 randomly selected non-responders, who met the inclusion criteria, were contacted by phone to repeat the questionnaire. Telephone interviews were performed by a clinical trial assistant with the original questionnaire. As VAS scores are not designed for telephone interview, a verbal instruction was given to obtain a score of 0–100 for studied symptoms to best replicate the effect of the scale.

### Statistical analysis

Discrete data were analysed and presented as frequencies and % frequencies, while continuous variables were mainly presented as mean with standard deviations. Pearson’s rho was selected as a correlation coefficient as VAS and SNOT-22 scores are following the normal distribution. VAS bronchial, VAS ocular and VAS sino-nasal were calculated as means of the corresponding category symptoms. Specifically, VAS bronchial score represented means of cough, tightness on the chest, shortness of breath and wheezing. VAS ocular score was obtained from itchy eyes and tearing, whereas VAS sino-nasal score from nasal blockage, headache/pressure on the face, loss of smell, postnasal drip, runny nose, itchy nose, and sneezing. IBM SPSS Statistics 23.0 (IBM Corporation, New York, NY, USA) was used for statistical analysis and a *p* < 0.05 was considered significant.

## Results

Of over 400 patients who met the inclusion criteria, a postal questionnaire was sent to 325 individuals of which 202 returned a filled questionnaire (response rate 62.15%). Twenty-two patients were excluded due to incomplete filling of the questionnaire and/or missing informed consent. A total of 180 patients completed the VAS symptom severity scores and the SNOT-22 questionnaire and were eligible for analysis. Of the 180 subjects analysed, 60% were male and 40% were female. The mean age of the studied population was 51.7 ± 16.6, ranged from 16 to 88. About 35.8% had known allergy, 28.7% had asthma, and 12.4% were current smokers. Among patients, 53.9% had CRSwNP, 46.1% had CRSsNP. Using the above mentioned VAS cut-off points for defining the level of control in CRS, 10% were classified as well controlled, 28.3% partly controlled, and 61.7% as uncontrolled (Table 1).

**Table 1:** Patients basic demographics, clinical characteristics, co morbidities, and level of control

*Demographics*		
Gender, n (%)		
Female	72	40.0%
Male	108	60.0%
Age, years (mean ± SD|min–max)		
Female	51.4 ± 17.4	18–88
Male	51.9 ± 16.2	16–88
Total	51.7 ± 16.6	16–88
Surgery, n (%)		
Yes	149	83.2%
Number of FESS (mean ± SD|min–max)	1.8 ± 1.0	1–5
Smoking, n (%)		
No	122	68.5%
Current	22	12.4%
Ex-smoker	34	19.1%
Smoking duration, years (mean ± SD|min–max)	15.7 ± 10.2	1–37
*Clinical characteristics*		
CRSsNP, n (%)	84	46.1%
CRSwNP, n (%)	96	53.9%
*Co*-*morbidities*		
Asthma, n (%)	51	28.7%
Allergy, n (%)	63	35.8%
*Level of control*		
Controlled, n (%)	18	10.0%
Partially controlled, n (%)	51	28.3%
Uncontrolled, n (%)	111	61.7%

**Fig. 1:**
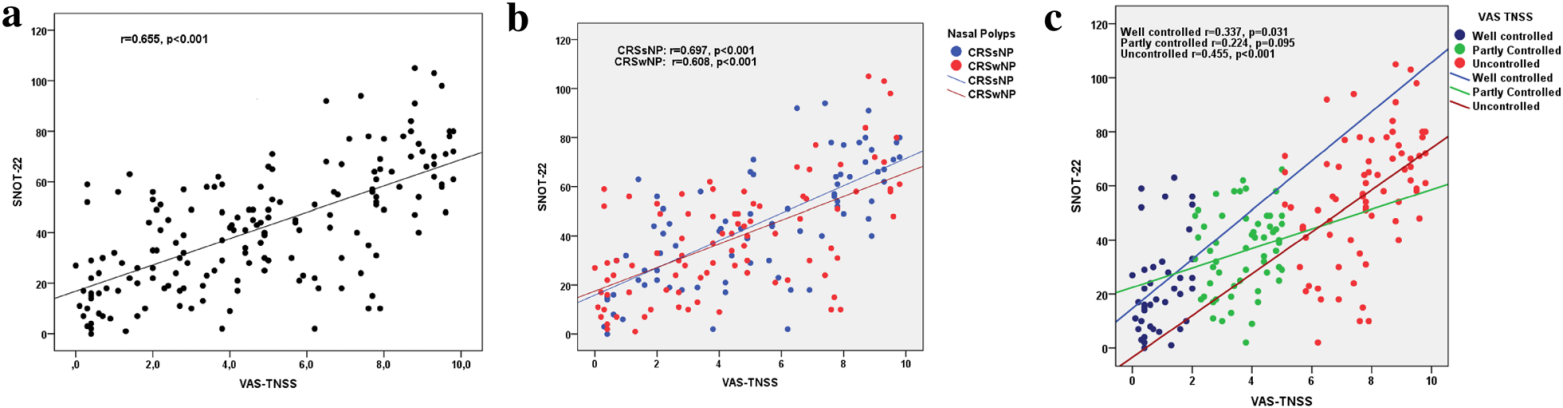
**a** Scatterplot of the correlation between SNOT-22 score and VAS-TNSS in CRS, **b** scatterplot in the two major phenotypes (CRSsNP, CRSwNP), **c** scatterplot of the correlation in different levels of disease control

A significant correlation between SNOT-22 and VAS scores for TNSS (Fig. 1a) and individual symptoms was found, with VAS-TNSS showing the strongest correlation, followed by headache/pressure on the face, postnasal drip, and obstruction (Table 2). The maximum value of Pearson’s rho test was estimated at r = 0.655, (*p* < 0.001) for VAS-TNSS, and the minimum value was r = 0.301, (*p* < 0.001) for VAS-Loss of smell score. Correlation between VAS scores and SNOT-22 remained statistically significant, irrespective of the distribution of VAS individual scores into subclasses based on symptoms origin (sino-nasal, ocular, bronchial). In this case, VAS for sino-nasal symptoms showed the strongest correlation with SNOT-22 (r = 0.738; *p* < 0.001), followed by bronchial (r = 0.683; *p* < 0.001), and ocular symptoms (r = 0.559; *p* < 0.001) (Table 2). Furthermore, we found VAS for TNSS to correlate significantly with SNOT-22 in both CRSsNP (r = 0.697; *p* < 0.001), and CRSwNP (r = 0.608; *p* < 0.001) phenotypes (Fig. 1b).

**Table 2:** Pearson’s correlation of SNOT-22 score with VAS scores in CRS patients

	SNOT-22	
	Pearson’s R	*P*
VAS-TNSS	0.655	< 0.001
VAS-blockage	0.499	< 0.001
VAS-headache/facial pressure	0.607	< 0.001
VAS-Loss of smell	0.301	< 0.001
VAS-postnasal	0.579	< 0.001
VAS-runny nose	0.472	< 0.001
VAS-itchy nose	0.353	< 0.001
VAS-sneezing	0.460	< 0.001
VAS sino-nasal	0.738	< 0.001
VAS-itchy eyes	0.470	< 0.001
VAS-tearing	0.552	< 0.001
VAS-ocular	0.559	< 0.001
VAS-cough	0.549	< 0.001
VAS-tightness of chest	0.578	< 0.001
VAS-shortness of breathe	0.579	< 0.001
VAS-wheezing	0.459	< 0.001
VAS-bronchial	0.683	< 0.001

The maximum value of Pearson’s rho test was estimated at r = 0.655, (*p* < 0.001) for VAS-TNSS, and the minimum value was r = 0.301, (*p* < 0.001) for VAS-Loss of smell and SNOT-22 score

Subsequently, we intended to evaluate the association between VAS and SNOT-22 in different levels of disease control. A significant association was found for SNOT-22 and VAS-TNSS in well controlled (r = 0.337; *p* = 0.031) and uncontrolled (r = 0.455; *p* < 0.001) but not in partly-controlled (r = 0.224; *p* = 0.095) CRS patients (Fig. 1c). No significant differences were found in outcomes of the 20 non-responders to our questionnaire compared to those who responded.

## Discussion

To date, SNOT-22 is used in different research fields and in clinical practice to determine the burden of disease, the outcome of medical or surgical intervention, and to improve candidate selection for surgery [8, 9]. However, a large number of CRS patients primarily visit their pharmacist or general practitioner to seek advice for their disease or they self-medicate. It is of vital importance to use a simple and reliable tool that can be used by all healthcare providers and patients for self-assessment [16, 18]. As such, VAS has been incorporated into different mHealth tools. Emerging technologies could increase patient participation in treatment decision-making [19]. Consequently, these new tools might improve compliance to treatment, increase level of control, and facilitate doctor-patient communication [19]. Recently, VAS was validated to assess allergic disease control on smartphone screens for the MASK-rhinitis project [14].

We found a significant association between SNOT-22 and VAS for all symptoms (*p* < 0.001), with VAS for TNSS showing the best correlation (r = 0.655). Interestingly, nasal obstruction which is considered to have the highest average severity among patients with CRS, did correlate with SNOT-22 but headache/facial pressure, and postnasal drip showed stronger association. Although the importance of headache/facial pressure as a cardinal symptom in CRS has been questioned, it appears to have the second strongest association (r = 0.607). VAS for loss of smell was found to have the minimum value of Pearson’s rho test (r = 0.301). These findings, yet atypical, suggest that patients’ perspective of symptoms severity is the result of an interaction between many factors. Age, gender, socio-economic status, psychological profile, and other comorbidities may modify patients’ perception of symptoms burden appraisal. In line with this hypothesis, several studies in the literature demonstrated lack of correlation between subjective and objective measures assessing symptoms severity [20, 21].

The strong association observed between VAS for bronchial symptoms and SNOT-22 (r = 0.683; *p* < 0.001) is justified on the basis of current knowledge on upper and lower airways interaction [5, 22]. CRS and asthma are common manifestations of an inflammatory process within the contiguous upper and lower airways. Furthermore, it is well established that asthma and CRS frequently coexist, and treatment of one condition could alleviate the coexisting one [5]. Concerning association between allergy and CRS, there is conflicting data in the literature. Nonetheless, allergy testing and treatment remain an option in CRS [5]. Herein, we show a significant correlation between VAS for ocular and bronchial symptoms and SNOT-22. Our results could be explained as patients with allergic, non-allergic rhinitis, and asthma patients were not excluded from the study, and may further support the link between upper and lower airways as stated in the “concept of united airway disease” [1, 5, 22].

Interestingly, a significant association was found for SNOT-22 and VAS-TNSS in well controlled (r = 0.337; *p* = 0.031) and uncontrolled (r = 0.455; *p* < 0.001), but not in partly-controlled (r = 0.224; *p* = 0.095) CRS patients. As this was a postal questionnaire survey, applying the current EPOS criteria for defining the level of control was not feasible [1]. Utilization of specific VAS–TNSS cut-offs for level of control assessment was based on a previous real-life study, where VAS scores of CRS symptoms were compared for different levels of control according to EPOS criteria in 389 patients [15]. Recently, the same cut-off points were used in mySinusitisCoach, an app for patients with CRS developed by medical experts in the field, to assess the level of disease control [23]. The weak correlation observed in the intermediate intervals (VAS > 2 and ≤ 5) could be explained by the halo effect, which may be noticed when several items are to be evaluated with different types of scales [11].

In line with our hypothesis, VAS scores for TNSS and for individual symptoms correlate well with SNOT-22. Among different aspects, mean VAS scores for sino-nasal symptoms showed stronger association with SNOT-22 than with ocular and bronchial symptoms. Overall, VAS-TNSS can accurately predict disease severity, level of control, and burden of disease which is in accordance with the revised EPOS statement [1]. Our data confirmed that VAS for TNSS can be used as a first and easy system to evaluate the burden of CRS followed by the more extensive SNOT-22 questionnaire if more detailed analysis is required. The high response rate (62.1%) that was achieved and the fact that data obtained from telephonic interviews were fully in line with the written questionnaires, could allow us to overcome speculation for bias deriving from the nature of the study. Despite the selection of CRS patients being treated in a tertiary referral center, and the relatively small sample size, our data underline the value of a simple tool like VAS for TNSS. As VAS scores are nowadays being used in the novel digital Apps for allergic rhinitis but also CRS [24], our data support the use of VAS for evaluation of the disease burden in daily life.

## Conclusions

In conclusion, we here show that VAS scores can be used in CRS to evaluate the burden of disease. Undoubtedly, our data are paving the way for a simple evaluation of CRS disease burden. Taking into account that VAS can be easily digitized, it can play a key role not only in everyday clinical practice but also in mHealth tools designed to monitor disease activity in CRS patients.

## Additional file

**Additional file 1.** VAS section of the questionnaire.

## Abbreviations

CRS: chronic rhinosinusitis
QoL: quality of life
COPD: chronic obstructive pulmonary disease
SNOT-22: sino-nasal outcome test-22
VAS: visual analogue scale
ARIA: allergic rhinitis and its impact on asthma
RQLQ: rhinoconjunctivitis quality of life questionnaire
TNSS: total nasal symptom score
VAS-TNSS: visual analogue scale for total nasal symptom score
CRSsNP: chronic rhinosinusitis without nasal polyps
CRSwNP: chronic rhinosinusitis with nasal polyps
