# Supplementary material for: Visual analogue scale for sino-nasal symptoms severity correlates with sino-nasal outcome test 22: paving the way for a simple outcome tool of CRS burden
Source: Clin Transl Allergy. 2018 Sep 3;8:32. doi: 10.1186/s13601-018-0219-6 (PMC6120084; doi:10.1186/s13601-018-0219-6)

**Seriousness of the complaints:** Please draw a vertical line to the point that best corresponds to, how bothersome were the following symptoms within the last month, as given to the example:

* Example None More than I can imagine


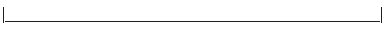


* Total sinus symptoms:

None More than I can imagine


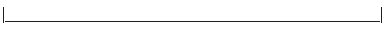


* Nasal blockage: None More than I can imagine


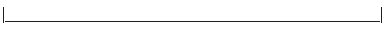


* Headache /pressure on the face: None More than I can imagine


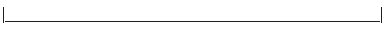


* Loss of smell: None More than I can imagine


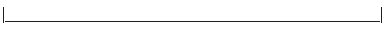


* Post-nasal drip (secretions from the nose down to the throat):

None More than I can imagine


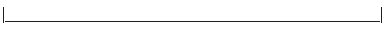


* Runny nose: None More than I can imagine


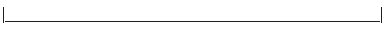


* Itchy eyes: None More than I can imagine


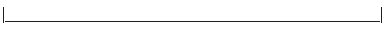


* Itchy nose: None More than I can imagine


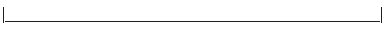


* Sneezing: None More than I can imagine


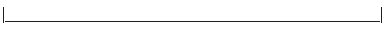


* Tearing: None More than I can imagine
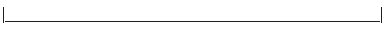


* Cough: None More than I can imagine


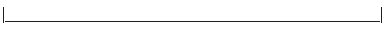


* Tightness/pressing sensation on the chest:

None More than I can imagine


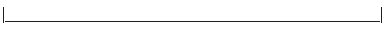


* Shortness of breath/difficulty with breathing:

None More than I can imagine


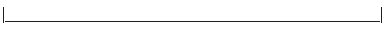


* Wheezing:

None More than I can imagine


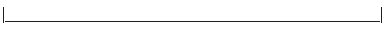

Supplement: Supplementary file 1 — Additional file 1. VAS section of the questionnaire. [file 13601_2018_219_MOESM1_ESM.docx]
